# The health system accountability impact of prison health committees in Zambia

**DOI:** 10.1186/s12939-018-0783-3

**Published:** 2018-09-24

**Authors:** Stephanie M. Topp, Anjali Sharma, Chisela Chileshe, George Magwende, German Henostroza, Clement N. Moonga

**Affiliations:** 1 Centre for Infectious Disease Research in Zambia, PO Box 30346, Lusaka, Zambia; 2 James Cook University, College of Public Health, Medical and Veterinary Sciences, Townsville, QLD, 4810 Australia; 3 ZCS Headquarters, PO Box 80926, Kabwe, Zambia; 4 University of Alabama at Birmingham, School of Medicine, Birmingham, AL 35233 USA; 5 Department of Global Health, University of Washington, Harris Hydraulics Laboratory, Box 357965, Seattle, WA USA

**Keywords:** Prisons, Health systems, Accountability, Health committees, Zambia

## Abstract

**Background:**

From 2013, the Zambian Corrections Service (ZCS) worked with partners to strengthen prison health systems and services. One component of that work led to the establishment of facility-based *Prison Health Committees* (PrHCs) comprising of both inmates and officers. We present findings from a nested evaluation of the impact of eight PrHCs 18 months after programme initiation.

**Methods:**

In-depth-interviews were conducted with 11 government ministry and Zambia Corrections Service officials and 6 facility managers. Sixteen focus group discussions were convened separately with PrHC members (21 females and 51 males) and non-members (23 females and 46 males) in 8 facilities. Memos were generated from participant observation in workshops and meetings preceding and after implementation. We sought evidence of PrHC impact, refined with reference to Joshi’s three domains of impact for social accountability interventions – state (represented by facility-based prison officials), society (represented here by inmates), and state-society relations (represented by inmate-prison official relations). Further analysis considered how project outcomes influenced structural dimensions of power, ability and justice relating to accountability.

**Results:**

Data pointed to a compelling series of short- and mid-term outcomes, with positive impact on access to, and provision of, health services across most facilities. Inmates (members and non-members) reported being empowered via a combination of improved health literacy and committee members’ newly-given authority to seek official redress for complaints and concerns. Inmates and officers described committees as improving inmate-officer relations by providing a forum for information exchange and shared decision making. Contributing factors included more consistent inmate-officer communications through committee meetings, which in turn enhanced trust and co-production of solutions to health problems. Nonetheless, long-term sustainability of accountability impacts may be undermined by permanently skewed power relations, high rates of inmate (and thus committee member) turnover, variable commitment from some officers in-charge, and the anticipated need for more oversight and resources to maintain members’ skills and morale.

**Conclusion:**

Our study shows that PrHCs do have potential to facilitate improved social accountability in both state and societal domains and at their intersection, for an extremely vulnerable population. However, sustained and meaningful change will depend on a longer-term strategy that integrates structural reform and is delivered through meaningful cross-sectoral partnership.

## Background

The Alma-Ata declaration on primary health care [1] advocated for a decentralised approach to the organization, delivery and management of health services [2]. According to this declaration, authority for planning, budgeting, managing and monitoring health activities would exist at the local level and would ensure citizen involvement in priority setting, implementation and monitoring. In part arising from the imperative to ensure such community involvement, a range of strategies designed to strengthen citizen engagement in health have emerged in the three decades since Alma Ata [2]. Of these, one of the most common strategies is that of community, neighbourhood or village health committees [3–7].

Zambia has a long history of decentralised health governance [8, 9] and ‘mainstream’ community engagement in healthcare via neighbourhood health committees [10, 11]. In the prison sector, however, things are different. Having only recently transitioned from a ‘prison’ to a ‘correctional’ paradigm, Zambian prisons’ recent history has been one of highly centralised and non-transparent operations, including health services planning and access. However, this began to change in 2010 with the appointment of the first Director of Health within the Zambian Corrections Service (ZCS) [12] and a new ‘open door’ policy that enabled non-government organisations to offer support in certain areas, including health care services.

### Zambian prisons

Zambia has a total of 87 prison facilities. Of these 54 are regular maximum or medium-security prisons, and 33 are open air low-security or ‘farm’ prisons for pre-release inmates. Zambian Prisons are severely overcrowded [13–16], the inmate occupancy rate (convicted prisoners and remandees) between 2014 and 2016 hovered around 270% of the official holding capacity of 6100 inmates. Previous and current work has highlighted lack of adequate food and potable water within prisons as direct and indirect contributors to the poor health status of both male and female inmates [17–19].

Of Zambia’s 87 correctional facilities, 17 have an associated health service. Eight of those 17 facilities have a health clinic within the prison walls established exclusively for inmate and officer use and staffed by health professionals employed ZCS. The remaining nine facilities have a Ministry of Health (MOH)-run primary health centre situated outside the prison but within walking distance. A total of 60 correctional facilities are dependent on a combination of prisoner-transfers to the closest MOH-run health facility or, occasionally, internal visits by MOH doctors [18]. In all prisons, a combination of lack of human resources for health, weak integration of health and security protocols and almost no oversight of prison-based health, contribute to sub-standard service access and quality [14, 17–20]. The profound isolation and lack of provision for basic needs make prison inmates in Zambia a highly vulnerable population.

### Zambian prisons health system strengthening project

From 2013, building on several years of partnership focused on prison tuberculosis control [20, 21] ZCS partnered with the Centre for Infectious Disease Research in Zambia (CIDRZ) to strengthen prison health systems and services [22]. The Zambian Prisons Health System Strengthening project (hereafter ‘the project’) included multiple components targeting the macro, meso and micro-level of the prison health system [23]. One component of this work included an 18 month consultation process regarding the conceptualisation, formalisation and establishment of male and female *Prison Health Committees* (PrHCs). Eleven intervention facilities including male and female holding facilities were purposefully selected for a first phase of PrHC establishment, which occurred between January and June 2014. Selection of the correctional facilities to be included was based on the largest and most overcrowded facilities. The process of selecting and training PrHC members and operationalising the committees was the culmination of an extended, two year consultation process involving ZCS, Ministry of Home Affairs (MHA), MOH and a range of NGOs. Key features of the committees included the co-membership of both officers and inmates; a remit for health promotion, service support and representation of inmate concerns as captured in a published terms of reference disseminated to all officers-in-charge (Table 1).

**Table 1:** Prison Health Committees (PrHC) Published Terms of Reference [49]

Every prison shall have a PrHC whose membership will comprise ZCS officers and inmates. The committee will be overseen by one prisons staff preferably the HIV coordinator. For purposes of adequately receiving and addressing inmates’ health concerns, the PrHC shall have a subcommittee comprising inmates and overseen by a ZCS officer preferably the HIV Coordinator. The Prison facility management (e.g. Officer In-Charge with authority from the ZCS-HD) shall have the authority to appoint those serving on the prisons health committees, including inmates who shall be selected from those with health backgrounds and/or those considered reformed/well behaved. However, inmates to serve on this subcommittee should be those that have received some training on health issues e.g. Peer Educators, HIV Counsellors. It is important for PrHC members to know that PrHC work is voluntary. Involvement in the PrHC will be unpaid, but will involve capacity building and participation in decision making related to facility-level health issues and services. Specific Duties: • Act as a link between inmates and the health centre/prison administration on health matters • Work together with other inmates to identify health problems in prisons • Work together with Prison Administration to address identified health concerns in prisons • Jointly coordinate health activities in prisons according to ZCS-HD directives • Provide a platform for discussion of health issues between inmates representatives and Prison Administration • Ensure that all inmates participate in prisons health activities • Collate and routinely report on health service information to ZCS-HD and ZCS HQ.	

Although contentious among some senior Ministry and Corrections stakeholders, a key driver of the formation of the PrHCs, was recognition by other senior government and non-government officials of the need to improve (within the constraints of a security setting) inmate representation in relation to health needs, and related to this, the accountability of facility-based staff for service planning, access and quality. Representation of this type may be seen as a type of ‘social accountability’ intervention; that is, a citizen effort to hold the government to account for the provision of essential services [24]. Although implemented in an unorthodox setting, the idea for PrHCs drew on experiences documented in the primary health care literature, which demonstrated that under the right conditions, health committees are capable of strengthening management and accountability of peripheral health services [3–7], as well as literature in the governance and accountability domain describing citizen ‘voice’ and ‘action’ interventions, such as community score-cards and social audit [25]. In this paper, we present findings from an evaluation of eight PrHCs, conducted 18 months after first formation.

## Methods

### Study Design & Procedures

Although this study was not designed as a realist evaluation, it was influenced by aspects of theory driven research, including a concern to understand ‘what worked’ ‘for whom’ and ‘under what circumstances’ [26]. Data collection was thus designed to help explore not only PrHC outcomes, but also the contextual factors and mechanisms that may have influenced those outcomes. [6, 7, 10, 27]. Contextual factors were defined as structural features – material or relational – that lay outside the influence of project design, but which influenced decisions and operations relating to it. While “mechanisms” were understood as underlying processes that operate in particular contexts, being typically invisible, sensitive to variations in context, and responsible for generating outcomes [28–30].

Qualitative methodologies including in-depth interviews, focus groups and observations, supported by document review were used to identify factors contributing to project successes and shortcomings. Reviewed documents included publicly available ZCS planning documents and publically documented priorities, plans and processes for health system strengthening. We additionally reviewed project documentation, including the project logic diagram, which provided a reference to better understand implementation fidelity and to evaluate success against the project’s own objectives. Complementary data were sought from in-depth interviews and focus group discussions at every level of the prison health system and are outlined in Table 2 and below.

**Table 2:** Data collection summary

Study population	Activity	Inclusion criteria^a^	Number of respondents
ZCS Headquarters	IDI	Employed by/seconded to ZCS	7
MOH / MCDMCH / MHA Officials	IDI	Currently employed by MOH, MCDMCH or MHA Involved with Zambian prison health services	3
NGO other community stakeholders	IDI	Current NGO or community member involved with prison health services	2
Facility Officers in Charge	IDI	Current appointment as the officer in charge (or acting) in a Correctional facility^b^	3 female 4 male
Appointed PrHC members	FGD	Appointed members of PrHC Able to provide informed consent	8 FGDs (Totalling 21 Females 51 Males)
Non-PrHC inmates	FGD	Inmate non-member of PrHC Able to provide informed consent	8 FGDs (Totalling 23 Female 46 Males)

^a^Exclusion criteria for all categories were a respondent < 18 years’ old and/or a known history of mental illness

^b^Officer in Charge for Facilities 5 and 6 – adjoining male and female prisons – was the same person

Participant (during meetings at which project staff were stakeholders) and non-participant observations were recorded in research memos as part of the ongoing project program monitoring. Based on extensive, typically hand written notes, research memos incorporating observations from prison-visit interactions, decision processes and relationship development related to prison health planning. Research memos were created as electronic files and coded for date, location and theme.

Funding constraints precluded sampling from all 11 facilities where PrHCs were initially established. We thus purposefully selected eight of the 11 facilities to achieve geographic spread (selected facilities fell across three of the four provinces represented in the larger sample), representation of administrative and security level (selected facilities covered District and centrally administered sites, and medium and maximum security sites) and gender representation (we selected sites that have female wings, which cumulatively hold the majority of female inmates in Zambia).

In-depth interviews (IDIs) were carried out with Corrections Officers, officials from MHA, MOH, and key stakeholders from civil society groups. Sampling was purposive and based on identification of respondents’ knowledge of, and involvement in, prison health governance or service delivery. The Officer-in-Charge or their delegate was interviewed at all eight sites. Among these key correctional officers, years of tenure in their current position was at, or just over, two years for five in-charges; 2 years for one and 15 years for another. Separate focus group discussions were carried out with PrHC members (comprising both inmates and officers who were active members of the PrHC) and non-members (inmates only). Focus groups were held in both male and female wings of all study facilities. Recruitment for focus groups was on a first come, first served basis with a minimum of eight participants in each case. In conjunction with the Officer-In-Charge, a study investigator issued an open invitation to attend one of two focus groups sessions – one for PrHC members and non-members respectively. Separate sessions were held for PrHC members and non-members in order to compare and cross-check their experiences and perceptions. Focus groups with PrHC members were hosted with officer and inmate members together. The Officer-In-Charge, or their delegate, at each of the eight facilities provided an overview of the study to potential participants and referred those who were interested to a room designated for the focus group. At the meeting, a multi-lingual Zambian study investigator provided more detail about the study, invited and answered questions, and asked if the participant(s) were still willing to participate. Verbal informed consent was then sought the language of the participant’s choice (*Bemba, English, Nyanja, Tonga*).

Interview and focus group question guides were specific to the type of participant. Participants were interviewed or participated in focused discussions for approximately one-hour. All interview and focus group participants permitted audio-recording for later transcription and analysis. No payments were made for involvement in any of the activities.

### Data management

All audio-recordings were transcribed directly into English in Microsoft Word™ along with expanded field notes. A research assistant fluent in all four languages compared the transcripts to audio-recordings and assessed them for accuracy, completeness and compliance with formatting requirements. Any anomalies were addressed by the interviewer or facilitator supported by field notes.

### Analysis

In a first phase of analysis, the principal investigator consulted the team to develop a thematic framework for data analysis using both inductive reasoning (focused on enabling contextual factors and catalytic mechanisms) and deductive reasoning based on Joshi’s three domains of impact for social accountability interventions [31]. Joshi’s domains of impact include: i) state responsiveness, ii) societal impacts, and ii) state-society relations. We interpreted ‘state responsiveness’ as encompassing the behaviours of any government official, down to, and including, frontline corrections staff. We narrowly interpreted ‘societal impacts’ as relating to either material or knowledge-based impacts on the inmate population. State-society relations were understood to encompass any type of inmate-government relationship, with a primary focus on facility-level interactions between inmates and corrections staff. A codebook was developed to capture data related to different types of PrHC outcomes in these three domains including material, relational and knowledge-based effects, as well as inductively identified contextual factors and supportive ‘mechanisms’.

Recognising the unusual setting and unique nature of this intervention, and with the aim of extending the generalisability of this work, a second phase of analysis was also carried out. Here we considered our first round findings through the lens of George et al.’s Dimensions of Accountability Framework [32] in order to better understand whether the already identified outcomes of PrCHS had a meaningful effect on *overall* prison health system accountability. The Dimensions of Accountability Framework synthesises and maps multiple dimensions of accountability onto three intersecting ‘axes’ – power, ability and justice – suggesting that all three are required to ensure sustained and authentic improvements in overall health system accountability. This framework was used to reflect on gaps and achievements of PrHCs in terms of promoting more sustainable, and sector-wide change in the prison health system in Zambia, thus providing a more universally comparable assessment of project impact.

### Challenges and limitations

Data for this study were largely collected by project staff, introducing the potential for positive-bias in outcome evaluation. In particular, we acknowledge the potential for desirability bias among respondents who may have been inclined to praise a project that brought funding or in-kind support. While outsourcing evaluation activities may have mitigated this problem, issues of trust and access to hyper security-aware stakeholders would likely have undermined our ability to conduct such an evaluation at all. In this instance, investigator involvement in the project was important both for the ability to access key stakeholders, as well as for the critical insight into the way project activities interacted with the broader context, highlighting the contingent, embedded and iterative nature of project impacts, in situ [33]. Due to ethical requirements, two authors in this manuscript were not investigators and had no access to raw interview or focus group data. They nonetheless played a critical role in facilitating project and study activities and provided important reflections on investigator-led analysis. As is necessary in implementation and evaluation research of this type, we engaged in careful and continual reflexive interpretation of project data, constituting an important risk mitigation technique along with systematic consideration and reporting of both impacts and challenges throughout.

### Ethical considerations

All project staff were trained in fundamental ethical principles and good research practices. The need to respect persons and their privacy were emphasized and constituted part of the Standard Operating Procedures. Inmate identifiers were not collected. All written and digital records were kept in a secured and locked area. All computer entry and networking programs were on password protected servers where data are encrypted. Analysis datasets were identified by study identifiers. Completed interview and focus group transcriptions, notes, and audio-recording are kept confidential. The University of Zambia Biomedical Research Ethics Committee and the University of Alabama in Birmingham Institutional Review Board approved the study.

## Results

In the following section we present findings on: i) important contextual factors that helped enable PrHC outcomes; ii) the challenges to, and impact of PrHCs in each of Joshi’s three domains of social accountability [31] and iii) the mechanisms that appeared to catalyse these outcomes. In the Discussion we further consider the sustainability, authenticity and depth of these outcomes with reference to George et al.’s Dimensions of Accountability Framework [32].

## Contextual factors

Across the different facilities, four major contextual factors were identified as creating an enabling environment for PrHCs to operate and flourish. Reflecting on the macro-level environment that had enabled the development of PrHCs in the first place, respondents highlighted inputs from CIDRZ, who convened and funded the consultation process and brought in other respected non-government partners (e.g. UNODC). Involvement of these partners and the repeated opportunities for otherwise siloed MHA and MOH officials to meet and discuss in a non-threatening environment, was described as promoting ‘outside-the box’ thinking in relation to Zambia’s profound prison health challenges. CIDRZ’s subsequent role in supporting the development of a PrHC training curriculum and resources for the initial training in the 11 start-up facilities was also noted as playing a critical contextual role.

The strong advocacy of the ZCS Commissioner General for the idea of PrHCs constituted a second important contextual factor, with multiple respondents noting that both his advocacy in the initial stages, and his subsequent issuing of a Central Directive to all correctional facilities were critical to the successful establishment of the new committees. In a profoundly hierarchical organisational setting, for instance, the Central Directive elevated the status of PrHCs in the eyes of facility Officers-in-Charge and ensured formation of the committees was seen as a priority by ZCS headquarters.

A third contextual factor supporting PrHC operation, was the high degree of buy-in from Officers-in-Charge described in most sites. As identified in the quote below, support for PrHCs among Officers-in-Charge was a critical prerequisite for both their establishment and subsequent operations, given the power these individuals wield in site-level operations. Without such support PrHCs would likely have existed on paper only:

*The Officer-in-Charge is the owner of everything here. So if he does not want something to happen, then for sure it cannot happen. But he is supportive of this committee. That is why we have seen them do well. (F4, Female, Non Member).*

A final contextual factor contributing to an enabling environment, was the alignment of PrHC functions with existing inmate hierarchy, which ensured more powerful inmates worked with (rather than undermined) PrHC decisions. Cell captains, for example, have authority to discipline other inmates through assignment of ‘punishment tasks’ or reporting them to officers for more serious transgressions. Cell captains are also typically responsible for managing and mediating inmates’ requests for access to health services [18]. The degree of direct involvement of cell captains varied from facility to facility but in a number of cases such involvement was described as an important facilitating factor:

*It’s such a nice composition of men and women that are in this committee, in the sense that not only do we have officers, three quarters of the members, if not all, are what we might call ‘Cell Captains.’ In short, they are leaders and being leaders, it’s very easy to sensitize or disseminate to fellow in-mates where issues to do with health are concerned (F7, Male Member, 9).*

## PrHC outcomes

### State domain – Impact on government actors

The formation of PrHCs had an impact on government actors in ZCS headquarters and MHA, challenging long accepted norms relating to inmates’ right to fraternise with officers and participate in or support health service planning. The development of the PrHC concept, and design and formalisation of a terms of reference was an iterative 18 month consultation process involving robust debate among ZCS, MOH, MHA and civil society representatives. A recurring concern reflected in meeting minutes from these consultations was that the formation of a committee comprising both inmates and officers would undermine the authority of correctional officers and thus overall security. Advocacy from senior members of the ZCS Health Directorate proved key in persuading those who were most opposed to the idea that it was indeed feasible; and a permanent rule change, codified in the published PrHC Terms of Reference was the outcome. As documented in several stakeholder meeting reports, the formation of PrHCs without incident and personal affirmations by Officers-in-Charge contributed to a shift thinking among high-level Ministry and Correctional officials, who in the latter phases of the (larger) project mooted further opportunities for involving inmates in actions to improve their own health.

Formation of the PrHCs and the authorisation to meet regularly also had an impact on facility-level officials in the eight intervention sites. Across the eight study sites, inmate members and non-members reported that, contrary to previous experience, correctional staff were more willing to listen to and act on inmates’ health concerns or lead by example when it came to delivering health education.

*The officers here are involved [more] than [my last prison]. They really go out of their way to help us; I have used the clinic like four times. They are so involved. In [the other place] they would even push you away. (F5, Male, Non-Member 11).*

*And the officers also are really leading by example. When the vaccines for elephantitis came they also [received it] in our presence, even the medicines for bilharzia they also drank. (F7, Male, Non-Member 12).*

Corrections staff and health workers who were PrHC members also described improved staff responsiveness to inmates’ health needs. As explained by the two officers below, this was partly related to an improved understanding of inmates’ experiences but also related to the heightened sense of responsibility associated with the relationships formed within the PrHC.

*The formation of the PrHC has really brought change, even in our working culture, because [previously] we were just waiting for patients to come; we were not involving people on the ground […] but [because of] the PrHC our coordination has really improved. (F6, Female Officer-Health Worker).*

*Personally I have matured since this committee came in. All those things that I used to see as challenges I now see hope in them. This committee has helped me grow such that all the things I used to see as problems I now see hope in them. (F3, Male Officer, Member).*

Respondents additionally described an impact on senior officials’ (e.g. Officers in Charge) understanding and thus responsiveness to ongoing environmental health concerns, such as the need to address rubbish removal, and improve water and sanitation in their facilities. In three sites (F3, F7, F8) improvements to environmental health measures were reported as a result of PrHC advocacy to senior officers:

*Over the past 4 months we made a decision that we improve drainage [in the bathroom]. Because we have had issues and this place used to flood. We requested through the PrHC and the Officer-in-Charge instructed the Department of Works and Supply here in prison that it should be done. (F3, Male Member).*

In Facility 7, PrHC members reported working with the Officer-in-Charge to sign a Memorandum of Understanding with the city council to re-initiate rubbish collection from the prison three times a week. Since the rubbish was being stored in heaps in the open-air spaces of the male prison this initiative had a direct positive impact on public sanitation for all male inmates:

*We have done a bit good in garbage disposal. Serious measures were taken to be disposing our garbage [frequently] through the council. (F6, Male Officer, Member).*

While modest in scope, a key feature of the above-described improvements was implementation without additional (donor or NGO-funded) support, demonstrating some success by the PrHCs in leveraging existing public resources to support inmate health. Notwithstanding this, the most frequently reported and fundamental challenge reported by senior stakeholders and PrHC members was the ongoing lack of resourcing for many basic functions within the facility. This issue lies largely beyond the control of facility-level Officers-in-Charge and speaks to the central challenge of addressing overarching deficiencies in Zambia’s financing of prisons, described in more detail elsewhere [12].

Inmates and staff in the eight facilities described the support and responsiveness of the Officers-in-Charge as both a pre-requisite to, but also enhanced by, the PrHCs:

*Without doubt the Officer-in-Charge of this prison has been important. We all know that if he wanted he could have been blocking all our decisions. The committee has scored a lot [and] the Officer-in-Charge has really contributed. (F3, Male Member).*

*I would say the officer-in-charge [has been] supportive; and without his support, there was not going to be any successes. (F8, Officer Member).*

Such a situation constituted an inherent weakness for PrHC whose inherent reliance on ‘champions’ within each facility left them vulnerable. This was demonstrated by the experience of committee members in one female prison, where the departure of an officer-member ‘champion’ stalled the achievements of the committee:

The formation of this health committee really started bringing change, [but] ever since Officer Faith (*name changed) left, not [so much]. Things started changing but now they have come to a standstill. (F2, Female Inmate Member 5).

### Society domain - impact on inmates’ knowledge

In the eight sites, both committee members and non-members reported feeling empowered by the PrHCs, via increased knowledge of health and their improved capacity to prevent illness or act to improve their own health.

*I am proud of the knowledge that I got in this committee. I used to think that you cannot stay in the same place with the people who are sick with HIV/AIDS but now I have changed […] I have learnt a lot. (F4, Female Member 7).*

*The coming of this committee has taught us a lot such that we now know the symptoms of these illnesses. (F7, Male Non-Member).*

Improved knowledge about certain conditions and preventive actions empowered inmates to be proactive about improving prison health conditions as was emphasised by members of several committees:

*Prior to being a PrHC member, if I saw, for instance, stagnant water, I always said: “Whoever is responsible for this job will do it.” But after becoming a PrHC member it actually made me realize that … If there is an outbreak it will affect me too. So in a nutshell, it has really brought a sense of responsibility [F5, Male Inmate Member 9).*

Empowerment was also derived from the collective and representative nature of the PrHC which enabled inmates to have a voice without the risks inherent in expressing individual views. Several Officers-in-Charge noted this as illustrated below:

*I would say it’s unique in the sense that inmates have representation [on the PrHC] in each and every cell in the prison; so they have information on what is going on [and] who is sick and that actually is unique in its own way. (F1, Officer-in-Charge).*

*What I know is that, the way the committee works, the communication and making of the decisions, it is collective […] [Inmates] are heavily involved in the making of the decisions. (F7, Officer-in-Charge).*

Of critical importance to the shift in senior officials’ opinion of the initiative, a number of correctional officers noted that the establishment of the PrHCs empowered inmates in ways that helped, rather than threatened, their own work:

*The PrHC has helped us positively, because prisoners make their own decisions. They talk to their own friends and try to counsel each other. (F2, Deputy Officer-in- Charge).*

*I have seen a dramatic change because [of] the cooperation that I am receiving from the inmates [...] We have seen a lot of illnesses reducing in this facility because of the inmates we are working with and the way they are relating with their friends. I never knew that an inmate would take care of their fellow inmates, people abandoned [even] by their relatives. (Facility 3, Male Officer, Member).*

A key challenge was nonetheless identified in the high turnover of inmates within the Zambian system which constituted a risk for committees due to loss of technical knowledge from PrHC training sessions, and the continual effort required on the part of the remaining members to re-train and reconstitute committees. Since to our knowledge, the terms of reference for the PrHCs were not widely disseminated among non-member inmates following the initial establishment, institutional knowledge of the committees’ roles and responsibilities was also not necessarily embedded in the wider and highly fluid inmate population.

### Society domain - impact on inmates’ service access and living conditions

Concrete improvements to inmates’ living environment and some improvements in access to, or quality of services further contributed to inmates’ sense of empowerment. A summary of these PrHC-initiated or contributory impacts is provided in Table 3. Outcomes common to more than one site included routine PrHC symptom screening and monitoring for TB – strengthening the consistency and improving the availability of that service; provision of counselling for those on TB and HIV medications; and delivery of weekly health education lectures.

*One of the biggest successes has been the introduction of a TB register in the cells. In the past during TB screening in the morning, one of our fellow inmates would just walk in a cell and ask “How many have not gone for screening?,” and based on how many will raise their hands, you would leave out some. So the chances of having one person with TB hiding would be very high. But the introduction of the registers in these cells has made us as committee members register every person […] We don’t even need to stand and ask “How many have not gone for TB screening?,” because all you have to do is go to your register. (F6, Male Inmate Member).*

*The dissemination of information, we never used to work like this. But now we have a lot of peer educators and they are working hard in terms of sensitizing the inmates and even the work the inmates and the officers are doing is good. (F3, Male Inmate Member).*

**Table 3:** Improvements in behaviour, services, environmental conditions & monitoring

	Improved Service / Environmental Conditions	F1-M	F2-F	F3-M	F4-F	F5-M	F7-M	F8-M	Total
1	PrHC support to prison clinic: counselling, health education, TB symptom screening	1		1		1		1	4
2	HIV counselling and accompanied referrals		1		1				2
3	Adherence support for inmates with chronic conditions		1		1				2
4	Prompted Officer in Charge to commit engaging Works Department to unblock drains in inmate bathrooms			1					1
5	Improved notification / upward reporting of health issues to Office in Charge			1		1	1	1	4
6	PrHC led ban on indoor smoking enforced by senior (Special Stage) inmates			1					1
7	Advocacy to ensure inmates’ receive timely follow-up visits to clinic				1				1
8	Prompted local council to restart rubbish collection improving general hygiene Improved notification / upward reporting of health issues to Officer in Charge						1		1
9	New health and safety measures for food storage and rubbish removal							1	1
	Total	1	2	4	3	2	2	3	17

F6-F: None recorded. Non-PrHC members noted lack of knowledge of the group / its purpose

Improvements in access to basic health information and in some cases health services were also reported in several sites, as described by one female inmate below:

*There are a lot of things. I will tell you that before this committee came, inmates used to suffer a lot because the people at the clinic used to take forever to see them. But when this committee came, it made things easier for the inmates as they received a lot of information on prevention issues. So they now know how to prevent themselves from these illnesses. (F4, Female Inmate Member 8).*

Respondents also noted, however, a general lack of skills required to operationalise the health monitoring and reporting duties outlined in PrHCs’ terms of reference (Table 1), merging with the lack of experience of either inmates or officers in collecting or analysing routine health information. As a result, the material impacts identified in this evaluation were largely the result of opportunistic assessment, while long term monitoring of health or service trends remained weak.

Further, in one female prison (F6), inmate non-members reported limited awareness of the PrHC and listed few benefits of the committee’s operations. A key difference in the composition of this committee was the appointment of three female inmates to sit on the larger male PrHC formed in the adjacent male prison (F5) rather than formation of a standalone committee. The rationale for this decision was ostensibly to ensure that female committee members were aware of larger decisions and opportunities linked to the (better resourced) male prison. However, lack of representation of female officers from F6, and the limiting of inmate representatives appeared to undermine its operation as continuity and reach became a problem.

*This health committee, they should include more people to work. When they add more people, it may be easier […] There are a lot of us, [we need more than] two or three. (F6, Female Non-Member, 4).*

### State-society domain - impact on inmate-officer relationships

By providing a sanctioned channel for information sharing and decision making, PrHCs were described by almost all inmates and officers in this study as improving inmate-officer relations:

*Yes, decision-making is really unique in the sense that when we are meeting, we don’t count to say: "this is an officer, this is a [such and such]," we all treat each other as members. Each suggestion or every observation that has been brought forth is discussed accordingly. And if there is a decision, it is supported by each and every member, thank you very much. (F7, Male Inmate Member 2).*

In several cases, respondents indicated that PrHC-related interactions had deepened the relationship to the point that traditional power dynamics of the prison setting were less prominent:

*Things have changed. At the time [the PrHC] just started, we were not writing reports like we are doing now. We never used to go deeper in the way we were doing things. Even the communication we had with the officers was not good as it is now. You know, […] we check on each other and if we see that the officers are relaxing we tell them, and also if they see that we are relaxing they tell us. Together we make sure that work is done in prison. So things have changed from when it started. There are a lot of follow ups. (F4, Female Inmate Member).*

Importantly, even non-members recognised that PrHCs constituted a potential new channel to interface with prison officials in order to achieve health-related ends:

*Here I can say I see that convicts and officers are working together. It is something good. Because it’s easy for convicts to relate to their [inmate] friends. They can know my [health] weaknesses. Now the problem is: How they can help me quickly [if I am sick]? So on my side, I think [the PrHC] has become a good thing for the prisoners to work together with officers [to be able to help quickly]. The work has become easy to finish our illnesses. (F7, Male Non-Member).*

*Let’s say the toilets are supposed to be cleaned. Now as an in-mate, where am I going to get the [cleaner] to put in the toilet? So I will tell the health committee inmate who is more superior than me and who is closer to the officer: “We need this [toilet cleaner] and maybe we also need gloves. Can you tell this officer?” So they will go and see the people, and if they have some, they will give us. (F5, Male Non-Member).*

## Mechanisms Catalysing PrHC outcomes

Study data pointed to three mechanisms as important catalysts for the positive outcomes described above. Respectively these were: the emergence of productive communication between inmates and officers (state-society communication); strengthened trust between officers and inmates; and co-production of positive outcomes.

### Productive state-society communication

Formation of the PrHCs strengthened communication between inmates and correctional staff in several respects. First, the committees constituted a uniquely ‘safe space’ (legitimised at the level of ZCS headquarters as well as by facility Officers-in-Charge) for inmates and correctional staff to interact.

*The inmates and the officers, especially us the officers, sometimes we used to be shy to come out in the open because we are officers. But thank God, there are inmates who are helping officers to come out in the open freely so that they can know and help each other. (F4, Female Officer Member 4).*

*It is actually a good thing that [the PrHC] consists of inmates and officers because if it was only for inmates, it could have been difficult for us to meet just as inmates. We would be cited as people who would incite illegal activities. (F8, Male Inmate Member 4).*

As indicated by the officer and inmate quotes above, prior to the formation of the PrHCs any kind of meeting between inmates and officers was viewed as suspicious and likely to reflect badly on both parties. Socialisation of incoming ZCS officers, for example, was described by respondents as a process that repeatedly emphasised the untrustworthy nature of inmates and the need for vigilance against potential security risks. Inmates too were actively discouraged from talking to officers, with the right to approach an officer reserved for privileged or senior inmates only (e.g. cell captains) and even then only on certain matters. The formation of PrHCs and their sanctioning by ZCS headquarters thus provided a unique opportunity for approved exchange between inmates and officers.

Furthermore, the authority of PrHCs to both report and recommend actions to improve prison health enabled (potentially for the first time ever) a direct channel of communication between inmates and the Officer-in-Charge. As noted by two Officers-in-Charge below, the committees served a new and important source of information on existing or emerging problems as well as recommendations about how to address these:

*[The PrHC] is effective. It is highly responsive to any matter arising that is related to health. What I mean by being responsive is that, as we sit in the offices here, … We are now not worried that there will be a health issue that will arise that we won’t know about. We will now hear through the committee. (F8, Officer in Charge).*

*If I can call it as a bottom up approach to decision making because they are the ones on the ground to bring out issues and recommend courses of action which [I am] able to consider and approve. (F7, Officer in Charge)*

The state-society interaction facilitated by inmates’ greater access to lower and higher-ranking officers thus constituted an important mechanism influencing committee achievements.

### Trust

As referenced above in *Impact in State-Society Domain*, inmate and officer members of PrHCs in all eight facilities described PrHCs as helping to build perceptions of sincerity and fairness between the two main stakeholder groups:

*Concerning decision making it’s not just an officer who influences [it], no. It’s actually the whole group [who] decides. It’s not something that comes from one person. It’s about group work and the majority always wins. We sit down and talk […] and the unique part is the way we’ve been working with us inmates and officers, that’s one unique part. (F1, Male Member 3).*

Foundational to these strengthened relationships was improved trust between the two groups arising from mutual understanding and experiences and repeated positive interactions:

*With the formation and the existence of the prisons health committee we have also seen the relationship between the officers and the inmates being not as enemies, but as colleagues who can work together when it comes to health matters. We have also seen prison officers, even the Commissioner, address the inmates despite their rank. All this is because of the prisons health committee. [F8, Female Officer Member].*

Improvements in trust went in both directions. Inmates felt they could express needs and make proposals without being criticised for ‘speaking out of turn’. But officers also reported that they felt at ease sharing information and even seeking support from inmates. Indeed, in several facilities, shifts in officer attitude appeared to go beyond improved trust, to transform profound social prejudices with positive knock-on effects in terms of their approach to inmates’ health.

*Before […] there was a phrase they used and it goes: “An inmate is a snake.” But now we are considered to be human beings and when you are sick, they will consider you to be a patient. You will receive all the patient’s human rights, thank you so much. (F8, Male Inmate Member 4).*

### Co-production

As demonstrated in other settings [34] improved communication channels and nascent trust can be leveraged in the co-production of interventions or activities to improve health services. Ostrom [35] defines co-production as the goods produced jointly by citizens and the government. In this study, both inmates and officers described interactive and inclusive processes as leading to identification of local (facility) health priorities, and decisions and actions to improve health knowledge, access to health services, and health service quality:

*These successes have been possible because the committee members are able to work together and through the regular meetings. They are able to see their successes and failures together so they improve. (F4, Female Officer 2).*

## Discussion

This study provides insight into the formation and impact of a uniquely representative body – PrHCs – in the Zambian prison system. The establishment of PrHCs occurred against the backdrop of a project which was seeking to strengthen national and sub-national prison health system governance [22, 36] with the aim of improving health service access and health outcomes for Zambian inmates. PrHCs were seen as the frontline embodiment of a broader effort to strengthen the responsiveness of the prison health system to inmate health needs, and the accountability of a range of stakeholders for the actions and decisions therein.

Findings presented above constitute a largely positive picture of PrHC impact. Yet the prison setting is one of profoundly skewed power dynamics in which ‘citizen’ inmates are not empowered to claim basic rights in the same way as those on the ‘outside’. In the following discussion, therefore, we seek to map the empiric findings of this evaluation onto George et al.’s [32] Dimensions of Accountability framework in order reflect more deeply on the authenticity and sustainability of accountability impacts brought about by PrHCs, and their consequent ability to drive or contribute to ongoing improvements in health system coverage and quality. As noted, this framework brings together multiple dimensions of accountability organised according to three axes: the axis of power, necessary to spark change; the axis of ability, necessary to support change; and the axis of justice necessary to steer change.

### Axis of power

George et al. describe this axis as catalysing health system change by producing sticks that curb the potential abuse of power or neglect of duty, but also by offering ‘carrots’ that motivate the constructive agency of service deliverers [32]. Findings from this study suggest that there was some, albeit very limited impact of PrHCs on this axis. Profoundly skewed power dynamics of the prison setting meant that patronage of the committees was needed at both the central and decentralised level simply to enable them to exist. As described in the findings, the impact of the PrHCs was viewed by all involved as highly contingent on the backing of officers-in-charge, whose own responsiveness was heavily informed by the level of interest and engagement of the Commissioner of Prisons. Impacts on this axis - such as improvements in the maintenance of basic living environments or access to (already existing) health services - were largely limited to drawing attention to localised (and non-personnel specific) instances of neglect of duty, and importantly were not actions that were perceived to threaten existing power dynamics.

### Axis of ability

The axis of ability is described by George et al. [32] as supporting health system change by enhancing formal rules, responsibilities and standards that expand service deliverers’ authority to act, as well as the informal norms and inputs that support a change in performance. In this domain, PrHCs were more impactful. The provision of a published terms of reference that was recognised at both central and facility level constituted an important change of rules that provided a formal basis for PrHC members to meet and advocate for (local level) changes. Both PrHC and non-PrHC members in this study described PrHCs as a new institutional channel through which such expression and representation could occur. While acknowledging the differences from ‘mainstream’ services, some of these findings mirror aspects of similar interventions in non-prison settings, where the formation of representative bodies and/or the scheduling of routine interface meetings have enabled exchange and provided a platform for aggregating and expressing the citizen ‘voice’ [37–39].

Findings also indicated that inmate and officer co-membership in PrHCs strengthened trust between the two groups, with positive impacts on officers’ understanding of, and thus responsiveness to the health needs of inmates. Trust is theorised as dependent on assessment of competency, but also judgements of reliability, sincerity, generosity and fairness [40] and often linked to repeated (positive) interactions over time that provide a basis for such judgements to be made. In this study setting the shifts in both the formal authority to recommend change and the norms surrounding inmate-officer communication, decision-making and support constituted a meaningful shift on this axis of ‘ability’ in the prison setting; however the data provided limited evidence of deep changes in this ability at a higher administrative or policy-making level.

### Axis of justice

This axis is seen as steering the strategic direction of change in a health system by balancing political representation, community ownership and social equity in support of progressive change, rather than capture by self-interests [32]. Review of the findings suggests mixed impact of PrHCs on this axis. On the one hand, PrHCs provided a unique opportunity for inmate representation, and improved ownership over an agenda of facility-level change, through joint advocacy and participation in health service support. However, the degree to which these changes improved equity within prisons is uncertain.

Studies of social accountability interventions in non-prison settings have found that the individuals who participate directly are more likely to be wealthier and more educated, and thus quite capable of advocating for their own – rather than broader population level – interests [37, 41, 42]. Our data did not capture specific instances of ‘elite capture’. Nonetheless, the opportunity for such in the Zambian prison settings is real. Recent work in Zambian male and female prisons, for example, has demonstrated a robust inmate hierarchy that frequently protects its own interests at the expense of other inmates [18, 19]. Transcripts from both officers and inmates in this study demonstrated how more educated inmates and cell captains were more likely to be selected for membership. While our data suggest that PrHCs to date have worked to improve conditions for the inmate population as a whole, the potential to use committees for perverse ends, including through gate keeping access to health services is an important consideration of these bodies’ contribution to accountability on the axis of justice. Moreover, the likelihood of such perversion of justice seems larger should disillusionment among PrHC members and the wider inmate population set in once the “low hanging fruit” of basic health promotion and health service access have been addressed, and where ongoing resource shortfalls preclude deeper and more meaningful change. While PrHCs appear to be serving an important function at the local level, therefore, the importance of a larger program of work that includes advocacy and accountability efforts at the policy-making centre of government is clear [3].

## Conclusion

Attention to prisoner health both in Africa [43] and globally [44, 45] has grown in the past decade, but far more is required. As outlined in this and previous studies both the scope and depth of need in the Zambian prison health system are profound [18, 19, 46]. Macro-level structural determinants including nationally underfunded correctional systems, lack of capacity for health planning and management, and the use of security agendas to block basic changes to prison health governance continue to mitigate against bringing living conditions, health service access and quality, and inmate health outcomes into line with internationally accepted norms [47, 48].

A key contribution of this work is to show that PrHCs do have the potential to facilitate improved social accountability in an environment otherwise stacked against responsiveness and participation. Yet caution is required and a better understanding of the factors contributing to both desirable and undesirable change as a result of such structures is critical. In this project, change appeared possible due to high-level buy-in for PrHCs by central and prison-based officers and prisoners alike. This buy-in ensured actors’ authentic engagement in processes addressing health issues, enabled the creation of democratic and safe spaces provided by PrHC and underpinned the responsive, flexible action to changing prison conditions and health needs. But nuanced and continually updated understanding of facility-level context including the role of Officers-in-Charge and PrHC dynamics in supporting change, as well as national level politics and legislative reforms, will be crucial to support democratic decision-making, authentic engagement and appropriate action. This requires further analysis of the power, ability and justice in prisons and for incarcerated populations.

## Abbreviations

HIV: Human immunodeficiency virus
MOH: Ministry of Health
PrHC: Prison health committees
TB: Tuberculosis
ZaPHSS: Zambia prisons health system strengthening [project]
ZCS: Zambia Corrections Service
